# Book Reviews

**Published:** 1913-02

**Authors:** 


### BOOK REVIEWS

Diseases of the Eyes: C. Devereux Marshall, F. R. C. S., London. University of London Press; Oxford University Press, American Branch, 35 W. 32, N. Y.; 303 pages, 95 illustrations, $3.75.

This is a well prepared, systematic treatise which emphasises no hobbies and, from the practical method of presenting successive subjects, is especially suited to the needs of the general practitioner or of one in the transition period from general to special practice. The expert ophthalmologist will also find it valuable.

Text Book of Ophthalmology, in the form of clinical lectures. Dr. Paul Roemer of Greifswald, translated by Dr. Matthias Lanckton Foster. The Rebman Co., N. Y.; 571 large pages, 186 illustrations in the text and 13 colored plates. Vol. 2, Diseases of the Lids, Injuries, Vitreous, Sclera, Lachrymal Organs, Orbit, Glaucoma, Strabismus, $2.50.

We have already reviewed the first volume. In scope and attention to detail, this work is cyclopaedic while the clinical method and elaborate illustration facilitate the work of the student. It is one of the most elaborate and thorough treatises that we have seen for some time in any branch of medicine.

Transactions of the American Surgical Association, Vol. 13, edited by Archbald MacLaren, Recorder. Printed for the Association by Wm. J. Dornan, Philadelphia; 758 pages, illustrated.

It is not without regret that we review a volume of this kind. Such transactions involve a considerable expense to the members, especially when of such limited numbers. This is a comparatively unimportant point, but so large a mass of literature of the highest grade ought to be published in mediums available to a larger audience.

